# A case–control observational study of insulin resistance and metabolic syndrome among the four phenotypes of polycystic ovary syndrome based on Rotterdam criteria

**DOI:** 10.1186/1742-4755-12-7

**Published:** 2015-01-16

**Authors:** Avin S Jamil, Shahla K Alalaf, Namir G Al-Tawil, Talha Al-Shawaf

**Affiliations:** Department of Obstetrics and Gynecology, College of Medicine, Hawler Medical University, P.O. Box 383–65, Erbil, Iraq; Department of Community Medicine, College of Medicine, Hawler Medical University, Erbil, Iraq; Women’s Health Research Unit, Centre for Primary Care and Public Health, Barts and The London Medical College, Queen Mary University, London, UK; Department of Primary Care and Public Health, Imperial College, London, UK

**Keywords:** PCOS, Rotterdam phenotypes, Insulin resistance, Metabolic syndrome, Hyperandrogenism

## Abstract

**Background:**

Polycystic ovary syndrome (PCOS) is associated with an increased risk of insulin resistance (IR), metabolic syndrome (MetS), impaired glucose tolerance (IGT) and type 2 diabetes mellitus (T2DM). Metabolic aspects of the four PCOS phenotypes remain to be fully defined. The aim of this study was to compare metabolic parameters and insulin resistance among the four PCOS phenotypes defined according to the Rotterdam criteria and to determine predictors of these complications.

**Methods:**

A total of 526 reproductive-aged women were included in this observational case–control study. Of these, 263 were diagnosed as a PCOS based on Rotterdam criteria and 263 infertile women with no evidence of PCOS were recruited as controls. Biochemical, metabolic and insulin resistance parameters were compared in the two groups and the frequency of MetS and IR were compared among the four phenotypes. Data were analyzed for statistical significance using Student’s *t*-test and one way analysis of variance followed by a post-hoc test (least significant difference). Chi-square tests were used to compare proportions. Univariate and multivariate logistic regression analyses were also applied.

**Results:**

IR was identified in 112 (42.6%) of the PCOS women and 45 (17.1%) of the control (*P* <0.001). There were no significant differences in the frequency of IR and MetS between the four PCOS phenotypes. Homeostatic model assessment for IR (HOMA-IR) ≥3.8 was the most common IR parameter in PCOS and control groups. Women with oligo-anovulation (O) and PCO morphology (P) had a significantly lower level of 2-h postprandial insulin compared to women with O, P and hyperandrogenism (H) phenotypes.

Logistic regression analysis showed that body mass index, waist circumference, triglyceride/high-density lipoprotein ratio (cardiovascular risk), HOMA-IR and glucose abnormalities (T2DM) were associated with increased risk of having MetS (*P* < 0.05).

**Conclusions:**

PCOS women with (O + P) show milder endocrine and metabolic abnormalities. Although, there were no significant differences in IR, MetS and glucose intolerance between the four PCOS phenotypes, women with PCOS are at higher risk of impaired glucose tolerance and undiagnosed diabetes.

## Background

Polycystic ovary syndrome is one of the most common endocrine disorders affecting women of reproductive age, and is frequently but not consistently associated with insulin resistance (IR) and compensatory hyperinsulinism (HI) [1]. More than 50% of women with PCOS are insulin resistant, with an estimated that they have a 5- to 8-fold increased risk of type 2 diabetes mellitus (T2DM) compared with age- and weight-matched controls [2, 3]. The pathogenesis of T2DM is critically affected by IR and B-cell dysfunction [4–6].

The prevalence of IR in PCOS patients ranges from 44 to 70% [7, 8]. This wide range may be due to several factors, including the heterogeneity of the diagnostic criteria for PCOS employed in these studies [7], the genetic background among the assessed population [9] and differences in the methods used for defining IR [7, 10]. The presence of chronic anovulation associated with higher androgen levels correlates with lower insulin sensitivity and higher prevalence of cardiovascular risk factors, such as IR, impaired glucose tolerance (IGT), T2DM, dyslipidemia and metabolic syndrome (MetS). However, the presence of two PCOS phenotypes identified according to the Rotterdam criteria—hyperandrogenism and polycystic ovaries with ovulatory cycles and anovulation and polycystic ovaries without hyperandrogenism—show little or no evidence of IR using surrogate markers [7].

It is still unclear whether milder phenotypes have the same metabolic and reproductive consequences as the more severe forms and therefore, whether all of these metabolic complications should be considered using strict screening procedures for those patients with milder phenotypes [11, 12]. Some studies suggest that the additional PCOS phenotypes introduced according to the 2003 Rotterdam criteria, particularly phenotype D (O + P), are characterized by less severe endocrine and metabolic abnormalities [12, 13]. In contrast, one study reported higher serum insulin levels in group B (O + H) and an insignificant increase in homeostatic model assessment of insulin resistance (HOMA-IR) scores among groups B (O + H) and D (O + P) [14]; however, this difference was lost after matching for age and body mass index (BMI) [15]. Thus, these observations suggest that higher insulin levels and HOMA-IR scores exhibit a stronger correlation with the higher prevalence of obesity in these PCOS women than the phenotypes per se [11, 16].

To our knowledge, this is the first study to investigate the prevalence of IR and MetS among the four phenotypes of PCOS according to the Rotterdam criteria in Erbil/Iraq. The aim of this study was to compare the differences in clinical, metabolic and IR parameters between PCOS, its four phenotypes and a control group.

## Methods

### Study subjects and design

This was a case–control observational study carried out on 263 women confirmed to have PCOS based on Rotterdam criteria and 263 infertile women with no evidence of PCOS as controls attending outpatient gynaecology and fertility clinic in Maternity Teaching Hospital, which is the only public maternity hospital in Erbil in the Kurdistan region of Iraq. Patients were recruited from 1st of April 2012 to 30th of June 2013. The study protocol was approved by the Scientific and Ethical committee at the College of Medicine, Hawler Medical University (Iraq). Informed consent was obtained from all women included in the study.

The diagnosis of PCOS was based on the Rotterdam-PCOS criteria [17]. According to these criteria, PCOS was diagnosed if at least two of the following criteria were present: oligomenorrhoea/anovulation (defined as delayed menses >35 days or < 8 spontaneous hemorrhagic episodes/year), clinical hyperandrogenism (hirsutism using modified Ferriman–Gallwey score of ≥8) or biochemical hyperandrogenism (total testosterone > 0.481 ng/ml) and polycystic ovaries morphology on ultrasonography (12 or more follicles in each ovary measuring 2–9 mm in diameter, and/or increased ovarian volume >10 ml^3^).

Women with PCOS were divided into four phenotypes, based on the presence of olig-anovulation (O), hyperandrogenism (H) and polycystic ovary morphology (P) : (i) Phenotype A (O + H + P), (ii) Phenotype B (O + H), (iii) Phenotype C (H + P) and (iv) Phenotype D (O + P).

The control group included 263 infertile women aged 18–39 year who attended the hospital during the period of study for investigation of fertility or gynecological disorders and who consented to participate in the study. The women in the control group had regular menstrual cycles (21-35 days), no evidence of hirsutism and no polycystic ovary morphology on ultrasonography. They included patients with male, tubal factor, unexplained infertility or other non-endocrine or related conditions.

The following exclusion criteria were applied: the presence of systemic disease that could alter insulin sensitivity (such as cardiovascular disease and diabetes mellitus); women on medication for ≤6 months prior to the study (including oral contraceptives, glucocorticoids, ovulation induction agents, and estrogenic or anti-androgenic drugs or any medication for dyslipidemia or anti-obesity drugs that could alter the patient’s clinical presentation or hormonal profile); women with other endocrinological abnormalities such as primary hyperprolactinemia, thyroid dysfunction, and Cushing syndrome, congenital adrenal hyperplasia and androgen producing neoplasm.

### Study protocol

Detailed histories (menstrual, fertility, hirsutism, acne, greasy skin, acanthosis nigricance, scalp hair loss or thinning and family history of T2DM) were taken from all patients included in the study. This was followed by a full examination (including general and pelvic examinations).

Body weight (kg), height (cm), waist circumference (WC), hip circumference (HC) and waist-to-hip ratio (WHR) were measured in all women. The BMI was calculated by dividing the weight (in kg) by the height (in m) squared to assess obesity. Systolic blood pressure (SBP) and diastolic blood pressure (DBP) were measured with a sphygmomanometer.

Blood samples for baseline measurements were collected after an overnight fast on day 2 or day 3 of the menstrual cycle in the control group and after a spontaneous bleeding episode in the PCOS group or randomly in the case of amenorrhea. The circulating levels of total testosterone, sex hormone binding globulin (SHBG) and insulin (fasting and 2-h postprandial) were measured by electro-chemiluminescence immunoassays (ELECYS 2010, Modular Analytics E170, cobas e 601, Roche Diagnostics, Mannheim, Germany). The intra- and inter-assay coefficients of variation for insulin were 0.7–1.5% and 2.4–4.9%, respectively (μU/mL × 6.945 = pmol/L).

Anti-Müllerian hormone (AMH) was assayed using the ultrasensitive an enzyme-linked immunosorbent assay ELISA (AMH Gen II ELISA, Beckman Coulter, 250 S. Kraemer Blvd, Brea, CA 92821 USA). Glucose, total cholesterol (TC), high-density lipoprotein cholesterol (HDL-C), low-density lipoprotein cholesterol (LDL-C) and triglycerides (TG) were measured using an enzymatic colorimetric method (BIOLABO REAGENTS SA 02160, Maizy, France).

On the same day as blood samples were collected, transvaginal ultrasonography was performed with a 6.5 MHZ frequency vaginal transducer (GE LOGIQ P3 Expert/Logic P3, probe destination E8CS/E8C, USA) and the volume of each ovary was determined, as well as the number of small follicles in each ovary.

Immediately after baseline blood sampling, an oral glucose tolerance test (OGTT) was performed in which glucose (75 g) was administered orally and serum glucose levels were determined at 0 and 120 min. A fasting plasma glucose (FPG) of <100 mg/dL (5.6 mmol/L) and 2-h glucose during OGTT <140 mg/dL (7.8 mmol/L) were accepted as normal values [18].

Categories of increased risk for diabetes (prediabetes) were defined as follows: impaired fasting glucose (IFG) was diagnosed when FPG was between 100 and 125 mg/dL (5.6–6.9 mmol/L) and IGT was diagnosed when the 2-h plasma glucose (2-h PG) value during a 75 g OGTT was between 140 and 199 mg/dL (7.8–11.0 mmol/L). Diabetes mellitus was confirmed by FPG ≥126 mg/dL (7.0 mmol/L), a 2-h PG value during a 75 g OGTT of ≥200 mg/dL (11.1 mmol/L), or a random PG concentration ≥200 mg/dL (11.1 mmol/L) in the presence of symptoms [18].

IR was diagnosed by the presence of one or more of the following: fasting plasma insulin ≥20 μU/ml, fasting glucose/insulin ratio ≤4.5, 2-h glucose/ 2-h insulin ratio ≤1 and a cut-off value of HOMA-IR ≥3.8. HOMA was calculated using the formula = fasting glucose (mg/dL) × fasting insulin (μU/mL)/405 [19].

The qualitative insulin sensitivity check index (QUICKI) was calculated using the following formula [20]: 1/[log fasting insulin (μU/mL) + log fasting glucose (mg/dL)].

Homeostatic model assessment was used to determine B-cell function (HOMA-B) calculated according to the formula [21]: (fasting insulin in μU/ml) × 20/ (fasting glucose in mmol/L – 3.5) μU/ml/mmol/L.

The Matsuda insulin sensitivity index (ISI) was calculated using the formula [22]: 10,000/*√*(FG (mg/dL) × FI (μU/mL) × G (mg/dL) × I (μU/mL) (FG, fasting plasma glucose; FI, fasting plasma insulin; G, mean plasma glucose concentration; and I, mean insulin concentration during the OGTT).

MetS was defined according to American Heart Association & National Heart, Lung and Blood Institute (AHA/NHLBI, 2005) updated ATP-III Third report of the National Cholesterol Education Program Adult Treatment Panel III (NCEP ATP-III) (National Institute of Health (NIH), 2007) guidelines [23], when three or more of the following criteria were present: WC ≥88 cm, serum triglycerides ≥150 mg/dL , high-density lipoprotein cholesterol <50 mg/dL or the use of lipid lowering medication, blood pressure (BP) ≥130/85 mmHg or the use of anti-hypertensive medication, or FPG ≥100 mg/dL.

### Statistical analysis

Data were analyzed using the Statistical Package for Social Sciences (SPSS, version 19). The Student’s *t-* test was used to compare means of two groups. One way analysis of variance (ANOVA) was used to compare between means of three or more groups. A post hoc test (least significance difference (LSD); performed after ANOVA) was used to determine the statistical significance of difference between two groups. A chi square test of association was used to compare between proportions. When more than 20% of the expected counts were less than 5, Fisher’s exact test was applied. Logistic regression analysis was used where the dependent variable was a binary categorical variable. Variables found (by univariate analysis) to be significantly associated with the dependent variable were entered into the regression model as independent variables. A *P*-value of < 0.05 was considered to indicate statistical significant.

## Results

A total of 590 women who visited the outpatient gynaecology and fertility clinic of the Maternity Teaching Hospital, Erbil in the Kurdistan region of Iraq between 1st of April 2012 and 30th of June 2013 were eligible for inclusion in this study; however, 17 PCOS patients and 17 control individuals declined to participate and a further 30 controls were excluded because of other endocrinological abnormalities.

Table 1 depicts the clinical and biochemical characteristics of the PCOS and control groups. Glucose metabolism profiles were significantly different between the groups (P < 0.05); the PCOS group had higher values for fasting insulin, 2-h glucose, 2-h insulin, QUICKI, HOMA-IR, fasting glucose/fasting insulin, HOMA-B, Matusda index, ratio of 2-h glucose to 2-h insulin, IR, and MetS than those for the control group. Lipid profiles also differed significantly between the two groups; the PCOS group had higher triglyceride, total cholesterol, low-density lipoprotein (LDL) levels and cardiovascular risk (TG/HDL) than the control group as shown in Table 1. The PCOS group was associated with a greater risk (2.5-times) of IR than the control group (*P* < 0.001).

**Table 1:** **The clinical, hormonal and metabolic parameters in patients with PCOS and control women**

Variables	PCOS(n = 263)	Control(n = 263)	***P*** -value
Age (years)	26.78 ± 4.95	29.02 ± 6.04	<0.001
Waist (cm)	99.29 ± 13.30	93.25 ± 12.05	<0.001
Waist-hip ratio (WHR)	0.91 ± 0.75	0.89 ± 0.69	<0.001
BMI (kg/m^2^)	31.08 ± 5.82	28.66 ± 5.04	<0.001
Systolic blood pressure (mmHg)	125.04 ± 11.94	123.25 ± 11.28	0.078
Diastolic blood pressure (mmHg)	82.21 ± 8.07	81.29 ± 7.43	0.178
AMH (ng/mL)	5.39 ± 2.02	2.49 ± 1.72	<0.001
Total testosterone (ng/mL)	0.44 ± 0.21	0.27 ± 0.13	<0.001
SHBG (nmol/L)	28.01 ± 22.09	41.08 ± 32.39	<0.001
**Glucose metabolism profile**			
Fasting insulin (FI) (μU/ml)	15.98 ± 10.12	10.75 ± 5.62	<0.001
2-h insulin (2-h I) (μU/ml)	93.79 ± 69.33	59.15 ± 39.24	<0.001
Fasting glucose (FG) (mg/dL)	90.96 ± 16.72	90.01 ± 12.30	0.458
2-h glucose (2-h G)(mg/dL)	126.44 ± 46.75	116.06 ± 28.56	0.002
QUICKI	0.37 ± 0.03	0.34 ± 0.03	0.001
Ratio of 2-h glucose to 2-h insulin	1.96 ± 1.35	2.58 ± 1.40	<0.001
Fasting glucose/fasting insulin	7.74 ± 5.24	10.61 ± 5.88	<0.001
HOMA-IR	3.67 ± 2.683	2.41 ± 1.42	<0.001
HOMA-B	227.99 ± 154.67	161.94 ± 105.27	<0.001
Matusda index	5.21 ± 3.75	7.19 ± 4.13	0.005
Insulin resistance (IR)	112 (42.6)	45 (17.1)	<0.001
Normal GTT	176 (66.9)	196 (74.5)	0.105
Prediabetes (IFG and/or IGT)	75 (28.5)	61 (23.2)	
Type 2 diabetes mellitus	12 (4.6)	6 (2.3)	
Family history of T2DM	131 (49.8)	104 (39.5)	0.022
**Lipid profile**			
Total cholesterol (TC) (mg/dL)	173.30 ± 34.86	165.29 ± 32.59	0.018
Triglycerides (TG) (mg/dL)	125.73 ± 87.01	96.73 ± 57.15	<0.001
HDL (mg/dL)	43.69 ± 9.68	45.22 ± 8.85	0.059
LDL (mg/dL)	81.55 ± 23.83	77.31 ± 21.87	0.034
Cardiovascular risk (TG/HDL)	3.17 ± 3.03	2.28 ± 1.61	<0.001
Metabolic syndrome (MetS)	141 (53.6)	86 (32.7)	<0.001

Values are presented as mean ± standard deviation or number (%). *P*-values were calculated using Student’s *t-* test and Chi-square test.

Table 2 shows that the fasting serum triglycerides, HDL, TG/HDL, glucose, 2-h glucose and HOMA-IR were similar in the four PCOS phenotypes; however, significantly higher levels of fasting insulin, 2-h insulin and HOMA-IR, lower glucose/insulin ratio (GIR) and QUICKI were observed in the four PCOS phenotypes compared with the controls (*P* < 0.001). Women with phenotype D PCOS had significantly lower 2-h insulin levels compared with women with PCOS phenotype A (*P* < 0.001) (Table 2).

**Table 2:** **The clinical characteristics and metabolic profiles in different PCOS phenotypes and control women**

Parameters	A n = 139	B n = 10	C n = 36	D n = 78	E-Control n = 263	***P*** -value
Age (years)	26.96 ± 4.67^b,e^	32.70 ± 2.71^a,c,d,e^	26.64 ± 4.86^e^	25.76 ± 5.19^e^	29.02 ± 6.03^a,b,c,d^	<0.001
Waist (cm)	100.85 ± 12.42^d,e^	103.50 ± 8.71^e^	96.86 ± 14.08	97.10 ± 14.5^e^	93.25 ± 12.05^a,b,d^	<0.001
Waist/hip ratio	0.92 ± 0.06^e^	0.95 ± .05^e^	0.91 ± 0.09	0.91 ± 0.08	0.89 ± 0.07 ^a,b^	0.001
BMI (kg/m^2^)	31.79 ± 5.79^c,e^	32.83 ± 4.83^e^	29.41 ± 5.67^a^	30.36 ± 5.88^e^	28.66 ± 5.04^a,b,d^	<0.001
Systolic blood pressure (mmHg)	126.37 ± 12.46^d,e^	129.50 ± 13.01	122.78 ± 10.92	123.14 ± 10.99^a^	123.25 ± 11.28^a^	0.043
Diastolic blood pressure (mmHg)	82.81 ± 8.82	84.50 ± 4.97	80.42 ± 8.31	81.67 ± 6.67	81.29 ± 7.42	0.214
Cholesterol (mg/dL)	176.70 ± 31.66^c,e^	177.40 ± 31.39	162.44 ± 27.11^a^	168.35 ± 42.30	165.29 ± 32.59^e^	0.015
Triglycerides (mg/dL)	130.37 ± 90.88^e^	144.50 ± 45.32^e^	105.61 ± 59.51	124.33 ± 93.93^e^	96.73 ± 57.15 ^a,b,d^	<0.001
HDL(mg/dL)	43.74 ± 9.70	42.78 ± 8.12	43.99 ± 9.96	43.59 ± 9.86	45.22 ± 8.84	0.450
LDL (mg/dL)	85.08 ± 24.79^c,d,e^	83.70 ± 20.90	74.05 ± 17.21^a^	78.44 ± 24.19^a^	77.31 ± 21.87^a^	0.010
TG/HDL (CV risk)*	3.31 ± 3.45^e^	3.44 ± 1.15	2.69 ± 1.95	3.12 ± 2.807^e^	2.28 ± 1.6^a,d^	0.001
Fasting glucose (FG)	91.29 ± 19.74	93.70 ± 16.02	87.58 ± 10.1	91.58 ± 13.04	90.01 ± 12.3	0.555
2-h glucose	129.45 ± 49.9^e^	128.90 ± 30.76	124.36 ± 53.95	121.73 ± 38.49^e^	116.06 ± 28.52^a,d^	0.022
Fasting insulin (FI)	16.86 ± 11.41^e^	18.52 ± 11.18^e^	14.51 ± 7.65^e^	14.76 ± 8.22 ^e^	10.74 ± 5.61^a,b,c,d^	<0.001
2-h insulin	103.63 ± 74.65^d,e^	101.91 ± 81.45^e^	93.60 ± 69.4^e^	75.29 ± 53.01^a,e^	59.15 ± 39.2^a,b,c,d^	<0.001
Fasting glucose/fasting insulin	7.30 ± 4.40^e^	7.21 ± 4.78^e^	8.14 ± 4.87^e^	8.46 ± 6.64^e^	10.64 ± 5.89^a,b,c,d^	<0.001
Ratio of 2-h glucose to 2-h insulin	1.86 ± 1.40^e^	1.56 ± 0.84^e^	2.04 ± 1.66^e^	2.15 ± 1.17^e^	2.58 ± 1.40^a,b,c,d^	0.001
Matsuda index	4.81 ± 3.449^e^	5.01 ± 4.19	6.02 ± 5.33	5.58 ± 3.28^e^	7.19 ± 4.13^a,d^	0.001
HOMA-IR	3.873 ± 2.99^e^	4.48 ± 3.27^e^	3.19 ± 1.86^e^	3.46 ± 2.29^e^	2.41 ± 1.42^a,b,c,d^	<0.001
HOMA-B	237.14 ± 163.32^e^	245.35 ± 139.7	227.93 ± 162.2^e^	210.21 ± 138.2^e^	161.93 ± 105.27^a,c,d^	0.001
QUICKI	0.32 ± 0.02^e^	0.32 ± 0.03^e^	0.33 ± 0.03^e^	0.32 ± 0.03^e^	0.34 ± 0.02^a,b,c,d^	<0.001

Values are presented as mean ± standard deviation. *P*-values were calculated using one-way ANOVA followed by multiple comparisons (post hoc test (least significance difference (LSD)).

*(TG/HDL) Triglyceride/ high-density lipoprotein (fasting), CV: cardiovascular risk.

^a^ *P* < 0.05 versus phenotype A PCOS, ^b^ *P* < 0.05 versus phenotype B PCOS, ^c^ *P* < 0.05 versus phenotype C PCOS, ^d^ *P* < 0.05 versus phenotype D PCOS, ^e^ *P* < 0.05 versus control.

(Note: (i) Phenotype A (O + H + P), (ii) Phenotype B (O + H), (iii) Phenotype C (H + P) and (iv) Phenotype D (O + P). A: Oligo-anovulation (O), + Hyperandrogenism (H), + Polycystic ovaries morphology (P).

Women with PCOS phenotype C presented with the same characteristics as those with classic PCOS (phenotype A), but in a milder form; this may represent a transition between the classic form and the control group.

After multiple comparisons and post-hoc analysis, women with PCOS phenotype D were found to demonstrate milder endocrine and metabolic abnormalities than those of women with the other phenotypes.

The Matsuda (insulin sensitivity) Index was significantly higher in the control group compared with the PCOS phenotype A and D groups.

Table 3 describes the elements that contribute to IR. All of the parameters that made up that contribute to IR were significantly greater in women with PCOS and in various phenotypes than in the controls (*P* < 0.001). Among the PCOS, PCOS phenotypes and control groups, similar results were obtained for glucose tolerance tests (P = 0.092) (Table 3).

**Table 3:** **Distribution of insulin resistance, glucose tolerance, MetS parameters and family history of T2DM among PCOS phenotypes and control**

Variables	PCOS (n = 263)	A (n = 139)	B (n = 10)	C (n = 36)	D (n = 78)	Control (n = 263)	***P*** -value PCOS phenotypes vs controls
Fasting insulin ≥20 μU/mL	64 (24.3)	33 (23.7)	4 (40)	10 (27.8)	17(21.8)	17 (6.5)	<0.001
Fasting glucose/Fasting insulin ≤4.5	61 (23.2)	32 (23)	4 (40)	10 (27.8)	15(19.2)	17 (6.5)	< 0.001
HOMA-IR ≥ 3.8	90 (34.2)	49 (35.3)	5 (50)	12 (33.3)	24(30.8)	36 (13.7)	< 0.001
Ratio 2-h glucose/2-h insulin ≤1	61 (23.2)	40 (28.8)	3 (30)	9 (25)	9 (11.5)	13(4.9)	< 0.001
Normal GTT	176 (66.9)	88 (63.3)	5 (50)	26 (72.2)	57(73.1)	196(74.5)	0.092
Prediabetes (IFG and/or IGT)	75 (28.5)	46 (33.1)	5 (50)	8 (22.2)	16(20.5)	61(23.2)	
Type 2 diabetes mellitus	12 (4.6)	5 (3.6)	0 (0)	2 (5.6)	5 (6.4)	6 (2.3)	
Insulin resistance (IR)	112 (42.6)	64 (46)*	5 (50)*	17(47.2)*	26(33.3)*	45(17.1)	<0.001
Metabolic syndrome (MetS)	141 (53.6)	81 (58.3)**	8(80)**	17(47.2)**	35(44.9)**	86(32.7)	<0.001
Family history of T2DM	131 (49.8)	65 (46.8)^+^	4 (40)^+^	18 (50)^+^	44(56.4)^+^	104 (39.5)	0.094

Data are shown as n (%). *P*-values were calculated using Chi-square test.

*Insulin resistance frequencies did not differ significantly between the PCOS phenotypes (*P* = 0.271).

**Metabolic syndrome frequencies did not differ significantly between phenotypes (*P* = 0.069).

^+^Family history of T2DM frequencies did not differ significantly between phenotypes (*P* = 0.525).

(Note: (i) Phenotype A (O + H + P), (ii) Phenotype B (O + H), (iii) Phenotype C (H + P) and (iv) Phenotype D (O + P). IFG: impaired fasting glucose, IGT: impaired glucose tolerance.

A higher proportion of women in the PCOS group presented with T2DM compared with that in the control group (4.6 vs 2.3%, respectively). This higher rate of T2DM was present predominantly in the phenotype D (6.4%), although the numbers were small to assess the statistical significance of this observation. The prevalence of prediabetes (IFG and/or IGT) was similar to that of T2DM between the groups.

There were no significant differences in the rates IR and MetS among the women with the different PCOS phenotypes; however, the lowest rate of IR was observed in the phenotype D, with this rate being approximately 33% lower than those in other PCOS phenotype groups. Furthermore, the highest rates of IR and MetS were observed in the PCOS phenotype B group.

Multivariable logistic regression analysis showed that BMI, WC, TG/HDL (CV risk), HOMA-IR and glucose abnormalities (T2DM) were associated with an increased risk of MetS (*P* < 0.05) (Table 4).

**Table 4:** **Logistic regression of metabolic syndrome as a dependent variable**

Independent variable	B	***P*** -value	OR	95% CI for OR	
				Lower	Upper
AMH	0.063	0.290	1.065	0.948	1.198
BMI	0.140	0.002	1.150	1.053	1.257
Waist circumference	0.066	0.001	1.068	1.028	1.110
TG/HDL (CV risk)	1.008	<0.001	2.739	2.170	3.458
HOMA-IR	1.414	0.001	4.114	1.823	9.282
Fasting insulin	-0.300	0.002	0.741	0.612	.896
2-h insulin	-0.004	0.134	0.996	0.990	1.001
Total testosterone	-0.765	0.303	0.465	0.108	1.997
**Glucose abnormalities**		0.024			
Impaired GTT	0.603	0.080	1.827	0.930	3.592
Type 2 diabetes mellitus Normal (reference)	3.574	0.025	35.641 1.000	1.553	818.185
Constant	-13.579	< 0.001	0.000		

OR: odd ratio; CI: confidence interval.

In logistic regression analysis using IR as a dependent variable, phenotype A (OR = 4.13; 95% CI for OR 2.602–6.567), B (OR = 4.84; 95% CI for OR 1.35–17.43) and C (OR = 4.33; 95% CI for OR 2.1–8.98) was shown to be associated with a four-fold increased risk of developing IR, while phenotype D (OR = 2.42; 95% CI for OR 1.37–4.28) was associated with only a two-fold increased risk in comparison to the control group (Table 5).

**Table 5:** **Logistic regression of insulin resistance as dependant variable and phenotype-control as independent variable**

Variables	B	P	OR	95% C.I. for OR	
				Lower	Upper
Phenotype-control		< 0.001			
A	1.419	< 0.001	4.134	2.602	6.567
B	1.578	0.016	4.844	1.346	17.432
C	1.467	< 0.001	4.335	2.091	8.984
D	0.885	0.002	2.422	1.370	4.282
Control (reference)			1.000		
Constant	-1.578	< 0.001	0.206		

Among the groups studied, PCOS phenotype A [OR = 2.874 (1.880–4.393 95%CI) and B OR = 8.233 (1.711–39.601 95%CI)] showed a highly positive association with MetS in comparison to control in logistic regression analysis (*P* < 0.001) (Table 6).

**Table 6:** **Logistic regression analysis for metabolic syndrome as dependant variable**

Variables	B	P	OR	95% C.I. for OR	
				Lower	Upper
Phenotype-control		<0.001			
A	1.056	<0.001	2.874	1.880	4.393
B	2.108	0.009	8.233	1.711	39.601
C	0.611	0.089	1.841	0.912	3.720
D	0.516	0.050	1.675	1.001	2.804
Control (reference)			1.000		
Constant	-0.722	<0.001	0.486		

## Discussion

The Rotterdam criteria for women with PCOS resulted in four phenotypes, which have dissimilar risks for IR and MetS. PCOS phenotypes with hyperandrogenism (A, B and C) have the worst metabolic presentations in terms of IR and MetS. Our findings also indicate that concurrency of these three PCOS phenotypes leads to increased severity of metabolic disorders, especially hyperinsulinemia, which may be related to more severe hyperandrogenemia (HA). All of the PCOS women in this study had higher triglyceride, total cholesterol, low-density lipoprotein (LDL), fasting insulin levels and HOMA-IR than the controls (which is consistent with previous reports [24]), and 2-h postprandial insulin [25].

Similar to our findings, a study conducted on 93 Polish women with PCOS, showed elevated serum concentrations of total cholesterol and LDL in the phenotype A PCOS compared with those with phenotypes C and D [14, 26].

In our study, phenotype B (O + H) PCOS showed the highest prevalence of MetS among the four PCOS phenotypes; these women were older and also had higher WHR, indicating a higher incidence of androgenic obesity and this higher prevalence is very likely to be related to higher BMI. These observations are in accordance with those reported by Tahrani et al. [26] and Welt et al. [27]. Women with phenotype A PCOS showed greater prevalence of MetS compared with those with phenotype C and the controls [28]. A comparison of PCOS patients diagnosed according to the Rotterdam, the Androgen Excess Society (AES) and the National Institute of Health (NIH) criteria conducted by Amato et al. [29] revealed that, regardless of the diagnostic criteria used, metabolic parameters and insulin sensitivity are critically important for the correct diagnosis and treatment of PCOS.

The prevalence of IR is higher among PCOS patients than that among control women (42.6 vs 17.1%); therefore, we further compared the hyperandrogenic and non-hyperandrogenic phenotypes of PCOS women in relation to IR and MetS. There were no significant differences in the prevalence of IR between the four phenotype groups [A (O + H + P) (46%), phenotype B (O + H) (50%), phenotype C (H + P) (47.2%) and phenotype D (O + P) (33.3%)]. Similarly, Kauffman et al. showed that insulin sensitivity indices were comparable across all PCOS phenotypes [30]. The many possible reasons for these inconsistencies include genetic variation between ethnic populations, environmental factors (including obesity) and the heterogeneity of PCOS phenotypes.

A comparison of markers of IR and endocrine characteristics between the different PCOS phenotypes conducted by Panidis et al. revealed that phenotype A (O + H + P) was associated with a higher prevalence of IR and more pronounced hyperandrogenemia than phenotype B (H + O). Phenotypes B (H + O) and D (O + P) with concurrent obesity were also characterized by IR. In contrast, phenotype C (H + P) was not associated with IR [11].

In accordance with our findings, Goverde et al. observed that the prevalence of metabolic disturbances and IR varies between different anovulatory PCOS phenotypes and reported that anovulatory hyperandrogenic women with phenotype A and B (with or without polycystic ovaries) had a higher prevalence of IR compared with phenotype D PCOS women and showed that WC was a predictor of MetS [31]. As another indicator of IR, our study adds that serum SHBG levels were significantly lower in all four PCOS phenotypes compared with those in the control group, which is consistent with previous reports [32, 27, 33]. Furthermore, both phenotypes A and B were more insulin resistant (greater prevalence) than the control, which has also been reported previously [33–35].

In some previous studies, no differences in fasting blood glucose levels were identified between the PCOS phenotypes and the control groups [27, 36], while another study showed higher blood glucose levels in H + O and H + P patients compared with control subjects [34]. Our data did not reveal any differences in serum glucose levels according to the 75 g GTT among the four PCOS phenotype, which is consistent with previous report [30].

In our study, although there were no significant differences in fasting insulin levels among the PCOS phenotypes, there were significant differences between the PCOS phenotypes and the control group. The present study also showed that 2-h postprandial serum insulin levels were significantly higher in the phenotype A than in the phenotype D PCOS, which is in agreement with the results reported by Chae et al. [37]. This may suggest that postprandial hyperinsulinemia plays an important role in HA and ovarian function in Iraqi women with PCOS.

As the glucose–insulin ratio decreases, the degree of IR increases. Shroff et al. reported the highest glucose–insulin ratio in the control group and the lowest ratio in the P + H + O phenotype group [34]. In our study, the lowest glucose–insulin ratio values were in the P + H + O and H + O phenotype groups, while the highest values were detected in the O + P phenotype and control groups, which is in accordance with the findings of Yilmaz et al. [24].

In a study comparing HOMA-IR scores, the highest values were detected in the P + H + O and H + O phenotypes and the lowest values were detected in the H + P phenotype and control groups [27], which is consistent with our findings. In another study, the highest HOMA-IR scores were associated with the H + P phenotype and the lowest with the O + P phenotype [34]. Chae et al. reported that there were no differences in HOMA-IR scores among the PCOS phenotypes, although there were significant differences between PCOS phenotypes and the control group, which is also in accordance with our results [37].

An increased risk of prediabetes (IFG and/or IGT) and T2DM in women with PCOS has been reported previously [38]. The present study showed a trend toward a higher risk of prediabetes (IFG and/or IGT) and T2DM associated with all PCOS phenotypes, compared with control subjects. These observations indicate the potential benefit of performing a 75 g 2-h OGTT in confirming glucose intolerance to inform the clinical prognosis and management of PCOS patients. The higher rate of T2DM in phenotype D PCOS may be related to the genetic factors because of higher percentage of family history of T2DM in this phenotype [39].

Using different surrogate markers of IR, we were unable to detect any significant differences in the prevalence of IR among the four PCOS phenotypes. Although we did not measure insulin sensitivity directly, a significant correlation between HOMA-IR and the insulin sensitivity index (gold standard euglycemic hyperinsulinemic clamp methods) has been identified in women with PCOS [40].

Logistic regression analysis revealed only a 2-fold increased risk of IR in phenotype D, while phenotype C had a 4-fold increased risk; these findings are similar to those reported by Svendsen et al. [41] who found that women diagnosed with phenotype C PCOS showed a higher degree of IR compared with those with phenotype D.

The numbers in group B (O + H) PCOS were small to assess the statistical significance and it’s regarded as the limitation of the study.

## Conclusion

In conclusion, our study conducted in a representative sample of the PCOS population, indicates that there is no significant difference in the prevalence of IR and MetS between the PCOS phenotypes, although there are significant differences between women with PCOS and non PCOS (control). Specifically, the prevalence IGT and undiagnosed diabetes is higher among women with PCOS. Women with phenotypes A and B exhibit the worst metabolic features. WC represents a clinically useful parameter for identifying anovulatory women with PCOS who should be screened for the presence of MetS.

PCOS women without HA showed lower 2-h postprandial insulin levels than PCOS subjects with HA. PCOS without HA could simply be a milder phenotype of PCOS, and may be associated with fewer metabolic complications. MetS is positively correlated with BMI, WC, TG/HDL (CV risk), HOMA-IR and glucose abnormalities (T2DM).
